# Imaging Glioblastoma With ^18^F-Fluciclovine Amino Acid Positron Emission Tomography

**DOI:** 10.3389/fonc.2022.829050

**Published:** 2022-01-31

**Authors:** Matthew L. Scarpelli, Debbie R. Healey, Shwetal Mehta, C. Chad Quarles

**Affiliations:** ^1^ School of Health Sciences, Purdue University, West Lafayette, IN, United States; ^2^ Barrow Neuroimaging Innovation Center, Barrow Neurological Institute, Phoenix, AZ, United States; ^3^ Ivy Brain Tumor Center, Barrow Neurological Institute, Phoenix, AZ, United States

**Keywords:** positron emission tomography, ^18^F-FACBC, fluciclovine, ASCT2, amino acid metabolism, PET imaging, glioblastoma, brain tumor

## Abstract

**Introduction:**

Conventional methods of imaging brain tumors fail to assess metabolically active tumor regions, which limits their capabilities for tumor detection, localization, and response assessment. Positron emission tomography (PET) with ^18^F-fluciclovine (fluciclovine) provides regional assessment of amino acid uptake in tumors that could overcome some of the limitations of conventional imaging. However, the biological basis of enhanced fluciclovine uptake is insufficiently characterized in brain tumors, which confounds clinical interpretation and application. This study sought to address this gap by correlating multiple biologic quantities with fluciclovine PET uptake across a range of human glioblastoma xenograft models.

**Methods:**

Thirty-one rats underwent orthotopic implantations with one of five different human glioblastoma cell lines. After tumors were established, fluciclovine PET and magnetic resonance imaging (MRI) scans were performed. The fluciclovine tumor-to-normal-brain (TN) uptake ratio was used to quantify fluciclovine uptake. MRI scans were used to assess tumor volume and gadolinium enhancement status. Histologic assessments quantified tumor cell proliferation, tumor cell density, and tumor cell amino acid transporters (LAT1 and ASCT2). Multivariate linear regression models related fluciclovine uptake with the other measured quantities.

**Results:**

Within the multivariate regression, the fluciclovine TN uptake ratio (measured 15 to 35 minutes after fluciclovine injection) was most strongly associated with tumor ASCT2 levels (β=0.64; P=0.001). The fluciclovine TN uptake ratio was also significantly associated with tumor volume (β=0.45; P=0.001) and tumor enhancement status (β=0.40; P=0.01). Tumor cell proliferation, tumor cell density, and LAT1 levels were not significantly associated with fluciclovine uptake in any of the multivariate models. In general, both enhancing and non-enhancing tumors could be visualized on fluciclovine PET images, with the median TN uptake ratio across the five tumor lines being 2.4 (range 1.1 to 8.9).

**Conclusions:**

Increased fluciclovine PET uptake was associated with increased levels of the amino acid transporter ASCT2, suggesting fluciclovine PET may be useful for assessing brain tumor amino acid metabolism. Fluciclovine PET uptake was elevated in both enhancing and non-enhancing tumors but the degree of uptake was greater in larger tumors and tumors with enhancement, indicating these variables could confound fluciclovine metabolic measurements if not accounted for.

## Introduction

Glioblastoma has a poor prognosis with median survival of approximately 14-20 months with standard of care treatment (1, 2). New techniques for assessing glioblastoma would be helpful for improving diagnosis, treatment planning, and response assessment. Currently, magnetic resonance imaging (MRI) is one the most widely used techniques for assessing brain tumors. Conventional MRI of glioblastoma provides qualitative assessments of tumor-induced edema (T2-weighted MRI) and blood-brain barrier breakdown (T1-weighted gadolinium enhanced MRI). These non-invasive assessments have proven useful throughout brain tumor patient care. However, these assessments are limited in that they only indirectly assess glioblastoma cells and they do not measure tumor aggressiveness (3). The utilization of quantitative functional or molecular imaging could help to overcome these limitations. For example, in solid tumors outside the brain, molecular imaging with ^18^F-fluorodeoxyglucose positron emission tomography (FDG PET) scanning is frequently utilized alongside other imaging modalities (4). FDG PET provides a direct measure of tumor cell metabolism that complements the measurements derived from anatomic imaging modalities such as computed tomography (CT) and MRI. FDG PET measurements have been shown to be more sensitive to malignant tumor cells than anatomic size assessments and FDG PET has the capability to provide an earlier indication of therapeutic effects (5, 6). Unfortunately, FDG PET assessments of brain tumors are confounded by glucose uptake in normal brain, indicating a need for a more specific molecular imaging technique in glioblastoma (7).

Amino acid metabolism is upregulated in brain tumors relative to normal brain tissues, providing a potential metabolic target for imaging. Studies have shown coordinated upregulation of the amino acid transporters LAT1 and ASCT2 in brain tumors that provide glutamine and essential amino acids to support tumor growth (8). Increased levels of these transporters has been associated with a poor prognosis in various cancers, including brain tumors (9, 10). These findings have led to the belief that these transporters are essential for the growth of some tumors and the development of therapies inhibiting these transporters (11, 12). The upregulation of LAT1 and ASCT2 has also been exploited for diagnostic purposes, including development of radiolabeled amino acids for PET imaging. ^11^C-Methionine (MET) and ^18^F-fluoroethyl-tyrosine (FET) are some of the most well studied amino acid PET radiotracers (13). However, these radiotracers are not widely available in the United States. ^18^F-Fluciclovine (fluciclovine) is an amino acid radiotracer that is widely available in the United States through commercial entities such as PETNET. Fluciclovine is already FDA-approved for the detection of recurrent prostate cancer but remains an experimental radiotracer for brain tumors. Fluciclovine PET provides high contrast images of brain tumors, indicating its potential for brain tumor imaging (14, 15). However, it has not been established whether fluciclovine PET uptake provides reliable quantification of brain tumor LAT1 and ASCT2 levels or whether fluciclovine PET measurements might be confounded by other physiologic processes, such as enhanced delivery due to blood-brain barrier breakdown (16). An improved understanding of the relationship between fluciclovine PET uptake and underlying brain tumor physiology would provide clarity on its potential role as a complementary imaging modality to conventional anatomic MRI.

The purpose of this study was to identify the biologic processes that most influence fluciclovine PET uptake in glioblastoma. *In vitro* studies have shown that fluciclovine is transported into brain tumor cells primarily *via* the ASCT2 and LAT1 amino acid transporters (17, 18). It has been assumed that these physiologic processes are similarly correlated with fluciclovine PET uptake *in vivo*. This study evaluated these assumptions across a range of patient-derived and immortalized glioblastoma xenograft models. Amino acid transporter levels, tumor cell density, cell proliferation, blood-brain barrier breakdown, and tumor volume are simultaneously assessed and related to fluciclovine PET uptake in this study. The goal of this study is to provide insight relating to what fluciclovine PET images are measuring and how they might be best utilized alongside conventional MRI scans to improve brain tumor care.

## Materials and Methods

### Animals and Disease Models

Two immortalized human glioblastoma cell lines (U87_luc_ and U251_luc_) were utilized in these studies (the tumor lines were transduced with luciferase, hence the luc subscript) (19, 20). In addition, three patient-derived xenograft (PDX) tumors harvested from fresh isolates of glioblastoma patients at Barrow Neurological Institute were utilized (GB7_luc_, GB126_luc_, and GB187_luc_). All tumor cell lines were provided by the Biobank Core Facility at St. Joseph’s Hospital and Medical Center and Barrow Neurological Institute and were deidentified and conformed to the Biobank Institutional Review Board’s protocol. All tumor cell lines were transduced with a lentivirus with luciferase to enable bioluminescent imaging and tracking of tumor growth using an *In Vivo* Imaging System (IVIS, PerkinElmer).

Before implantation, tumor cells were harvested and resuspended in PBS at a concentration of approximately 125 million cells/mL. Then 4uL of the cell suspension was orthotopically implanted into 5 weeks old immunocompromised RNU rats (Crl : NIH-Foxn1rnu) purchased from Charles River Labs (Wilmington, MA). Thirty-three rats were implanted with tumors (N=9 for U87_luc_, N=6 for U251_luc_, N=4 for GB7_luc_, N=6 for GB126_luc_, and N=8 for GB187_luc_) and underwent PET and MRI scanning. The time between implantation and imaging was 18 to 20 days for U87_luc_, 106 to 190 days for U251_luc_, 180 days for GB7_luc_, 26 to 28 days for GB126_luc_, and 62 to 90 days for GB187_luc_. The St. Joseph Hospital and Medical Center’s Institutional Animal Care and Use Committee approved of all experimental procedures performed in this study and all animals were treated humanely in accordance with the Laboratory Animal Welfare Act.

### PET and MRI Scanning

Tumor growth was regularly monitored with IVIS bioluminescence imaging. *In vivo* PET and MRI scanning was performed once IVIS bioluminescence signals averaged greater than 10^7^ photons/sec from tumor regions. PET scanning was performed using a Bruker Albira Si 3 ring preclinical PET scanner. Approximately 12 MBq of fluciclovine was injected intravenously at the tail vein for each rat. Dynamic PET scans were acquired from 0 to 55 minutes post-injection of tracer. For all PET scans the brain was positioned at the center of the field of view. An ordered subset expectation maximization algorithm was used for PET image reconstruction. Reconstructed PET images included corrections for scatter, deadtime, and decay of tracer.

MRI was performed with a 7-Tesla Bruker BioSpec preclinical MRI scanner. MRI scanning was performed immediately following completion of the PET scan. Animals were kept sedated when transferred between PET and MRI scanners and remained positioned on the same Bruker multimodality rat bed. To enable accurate co-registration between the MRI and PET images, a fiducial phantom filled with water and PET tracer was placed beneath the rat in the multimodality bed to act as a landmark (21). During MRI and PET scanning the rats were kept sedated using airflow of 1-1.5 mL/s with 1-3% isoflurane. The MRI scanning lasted approximately 45 minutes and included T2-weighted (T2W), T1-weighted (T1Wpre), and T1-weighted post contrast (T1Wpost) scans. The T2W scan included rapid acquisition with relaxation enhancement (RARE), sequence with a repetition time of 6,500 ms, an effective echo time of 50 ms, and a voxel size of 0.2 × 0.2 × 0.5 mm^3^. The T1W and T1Wpost scan included a Fast Low Angle SHot (FLASH) sequence with a repetition time of 16 ms, echo time of 2 ms, and voxel size of 0.2 × 0.2 × 0.5 mm^3^. Gadolinium contrast agent was injected intravenously between the T1W and T1Wpost scans.

PET and MRI images were registered using a rigid registration based on the fiducial phantom placed in the scanner bed (21). PET image voxels were corrected for injected dose and rat weight to give standardized uptake values (SUVs). Tumors were manually segmented using both the PET and MRI scans. Areas of abnormality on MRI and/or areas with visually increased uptake on PET were included in the tumor segmentations. A 10 mm^3^ region of interest (ROI) was placed in the contralateral brain to assess normal brain uptake of fluciclovine. The tumor-to-normal brain uptake ratio (TN) was calculated by taking the maximum tumor uptake (SUVmax) and dividing by the average contralateral brain uptake (SUVmean). Averaging of the dynamic PET frames provided early (0-15 minutes after injection), intermediate (15-35 min after injection), and late (35-55 min after injection) timepoint images. TN was extracted from each of the early, intermediate, and late timepoint images. Specific attention is given to the intermediate (15-35 minute) timepoint as that is most aligned with the recommend times for clinical PET scanning with other amino acid radiotracers in brain tumors (22).

### Pathology Assessments

All rats were sacrificed within 24 hours of PET and MRI scans *via* decapitation. Brains were dissected and immediately flash frozen in O.C.T. compound (4585, Fisher HealthCare) in a bath of 2-methylbutane (Arcos Organics) cooled with liquid nitrogen to between -40 and - 50C. Frozen sections (7-um-thick) were cut using a cryostat and mounted on Superfrost Plus slides that were stored at -20C until staining. The brain slices were stained to assess amino acid transporters (LAT1 and ASCT2) and cell proliferation (Ki67). Two slides from different coronal locations within the tumor were randomly selected for each staining with Ki67, ASCT2, and LAT1 (2 slides x 3 stains = 6 slides total per tumor).

### Chromogenic Immunohistochemistry

For LAT1 staining slides were fixed briefly in ice cold 4% paraformaldehyde in 0.1M PB (pH 7.4), washed in 0.1M PB (3x5minutes), washed in TBS (3x5minutes), incubated in 2.139% Sodium(meta)periodate in TBS for 20 minutes to block endogenous peroxidase activity, washed in TBS containing 0.1% Triton (3x5minutes), then incubated in TBS containing 0.25% Triton and 3% normal goat serum for 60 minutes for blocking and permeabilization. Next, the slides were incubated in primary antibody anti-LAT1 (Cell Signaling Technology rabbit #5347, dilution 1:50) in 0.1M PB containing 1% normal goat serum and 0.25% Triton-X overnight at room temperature. The following day slides were washed in TBS (3x5 minutes), and incubated in secondary antibody Goat Anti-Rabbit IgG, Biotinylated (Vector Laboratories, BA-1000, dilution 1:200) in TBS containing 1% normal goat serum for 60 minutes at room temperature. Following secondary incubation slides were washed in TBS (3x 5minutes) and incubated in avidin/biotin horseradish peroxidase complex (Vectastain Elite ABC labeling kit, PK-6100, Vector Laboratories) for 60 minutes at room temperature shielded from light. Slides were then washed in Imidazole Acetate Buffer (0.068g Imadazole, 0.68g NaAc Trihydrate in 100mL dH20, pH 7.4 w/glacial acetic acid) and HRP labeling was detected by incubating in 0.05% diaminobenzidine (DAB, Sigma D5637) with 0.015% H2O2 in un-pHed imidazole buffer for 20 minutes at room temperature. Following DAB incubation slides were washed and counterstained with hematoxylin (Hematoxylin 2, Richard-Allen Scientific, 7231) then dehydrated in graded alcohols and cover slipped using Cytoseal 60 (Thermo Scientific, 8310-4).

The stained slides were imaged using an Aperio Versa System with a 20x objective (Leica Biosystems). The entire coronal tumor region was manually segmented on each slide’s image using QuPath (23). The average DAB staining intensity was calculated for the tumor segmentation and normalized by the average contralateral brain DAB staining intensity. The measurements were repeated twice, once for each of the different coronal tumor locations assessed and averaged to provide an overall summary LAT1 measurement for each tumor.

### Immunofluorescence

For Ki67 slides were fixed briefly in ice cold 4% paraformaldehyde in 0.1M PB (pH 7.4) while for ASCT2 slides were fixed in ice cold acetone for 10 minutes. Both stains were then washed 6 times for 5 minutes each in 0.1M PB, followed by blocking and permeabilization in 0.1M PB containing 10% normal goat serum and 0.1% Triton-X for 2 hours at room temperature. All incubations were done in a humidity chamber to prevent evaporation. Slides were then washed in 0.1M PB (3x5 minutes) and incubated in either primary anti-Ki67 antibody (clone MIB-1, DAKO M7240, dilution 1:150) or anti-ASCT2 (D7C12, Cell Signaling Technology rabbit mAB #8057, dilution 1:500) in 0.1M PB containing 2% normal goat serum and 0.1% Triton-X at 4C overnight. The following day slides were washed again in 0.1M PB (3x5mins), incubated in a secondary antibody Alexa Fluor 488 Goat Anti-Mouse IgG1 (Invitrogen #A-21121, dilution 1:500) for Ki67 or Alexa Fluor 488 Goat Anti-Rabbit IgG (Invitrogen #A-11034, dilution 1:500) for ASCT2 for 3 hours at 4C, washed again in 0.1M PB (3x5mins), and mounted using Fluoroshield mounting medium with DAPI (Abcam, ab104139) to counterstain the nuclei.

Fluorescent images of the Ki67, ASCT2, and DAPI were acquired using an A1R HD25 confocal microscope (Nikon Instruments Inc). The entire coronal tumor region was manually segmented on each slide’s image using QuPath. Cell density (cells/mm^2^) within the tumor segmentation was quantified from the DAPI fluorescent images using QuPath’s automated cell detection algorithm counting from the blue channel. Similarly, the number of proliferating cells within the tumor segmentation was quantified from the Ki67 fluorescent images using QuPath’s automated cell detection algorithm counting from the green channel. The number of proliferating cells (Ki67) was divided by the total number of cells (DAPI) to give the proliferative index for each image. For the ASCT2 fluorescent image assessment, the average fluorescence signal intensity was calculated for the tumor segmentation and normalized by the average contralateral brain intensity. The cell density, proliferative index, and ASCT2 measurements were repeated twice, once for each of the different coronal tumor locations assessed and averaged to provide an overall summary measurement for each tumor.

### Statistical Analysis

Of the 33 rats implanted for this study, 31/33 were successfully measured with pathology and imaging for inclusion in the subsequent analysis. A univariate analysis was performed by calculating the Spearman correlation coefficients between PET uptake and each of the pathology measurements. A multivariate analysis was performed by calculating linear regression models with PET uptake as the dependent variable and having each of the pathology measurements included as independent variables. The tumor gadolinium enhancement status (binary variable derived from T1W MRI) and tumor volume were also included as independent variables in the multivariate regression. Before inclusion in the multivariate regression, measurements were log transformed (to better approximate normal distributions) (24) and scaled to have a mean of zero and standard deviation of 1 (to enable comparison of regression coefficients). The multivariate regression was performed using R version 4.0.2 using the Fitting Linear Models function. The partial regression plots for a given independent variable were formulated by 1) calculating the residuals of a multivariate model with the PET uptake as a function of all independent variables except the given independent variable of interest (e.g., notation used PET | Others), 2) calculating the residuals of a multivariate model with the given independent variable as a function of all the other independent variables (e.g., notation used ASCT2 | Others), and 3) plotting the residuals from 1 and 2 against each other (25).

## Results

**Figure 1:**
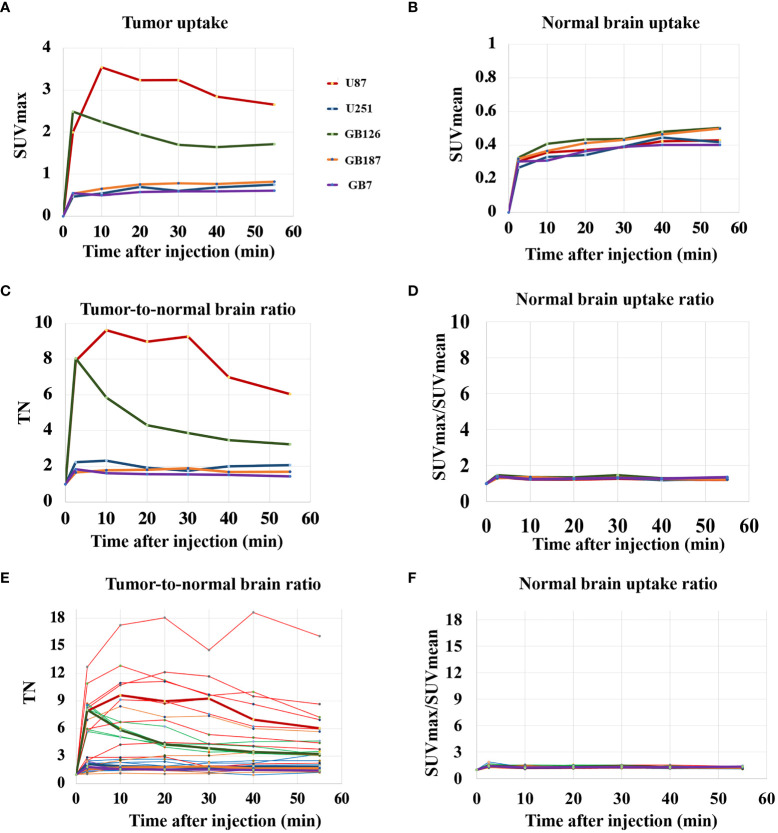
**(A)** PET uptake curves showing median tumor SUVmax for each tumor line. **(B)** PET uptake curves showing median contralateral brain SUVmean for each tumor line. **(C)** PET uptake curves showing median tumor-to-normal brain uptake ratio (TN) for each tumor line. **(D)** PET uptake curves showing the median normal brain SUVmax/SUVmean ratio for each tumor line. **(E)** PET uptake curves showing TN for all tumors, including the median lines for reference. **(F)** PET uptake curves showing the normal brain SUVmax/SUVmean ratio for all subjects.

**Figure 1** shows PET curves comparing median uptake values across the five tumor cell lines. In general, the U87_luc_ and GB126_luc_ tumors demonstrated rapid increases in tumor uptake that peaked within 15 minutes after injection. The U251_luc_, GB187_luc_, and GB7_luc_ tumors demonstrated a gradual increase in tumor PET uptake that continued increasing until the end of the scan (**Figure 1A**). The contra-lateral (normal) brain PET uptake gradually increased until the end of the scan for all tumors (**Figure 1B**).

U87_luc_ and GB126_luc_ tumors showed relatively high tumor-to-normal brain uptake ratios with median values fluctuating from 3 to 10 during the scanning time. U251_luc_, GB187_luc_, and GB7_luc_ tumors had relatively lower tumor-to-normal brain uptake ratios with median values fluctuating from 1.5 to 2 during the scanning time (**Figure 1C**). The contralateral normal brain SUVmax/SUVmean ratio had median values fluctuating between 1.2 to 1.3 across the tumor lines (**Figure 1D**). Tumor-to-normal brain uptake ratios varied the most across U87_LUC_ tumors (**Figure 1E**). Relatively little variation was found in the contralateral normal brain uptake when looking across all subjects (**Figure 1F**). In general tumors, could be visualized on fluciclovine PET images with the median TN_15-35_ across the five tumor lines being 2.4 (range 1.1 to 8.9). The exception was GB7 tumors, which had a median TN_15-35_ of 1.1 and no visible fluciclovine uptake in any of the tumors assessed (N=4). GB7_luc_ tumors also had no cells visually positive for ASCT2 on histology. For GB187_luc_ tumors, six out of eight (75%) were visualized on fluciclovine PET images. The two GB187_luc_ tumors that were not visualized on fluciclovine PET images also had the lowest ASCT2 staining intensities out of all GB187_luc_ tumors. For U87_luc_, U251_luc_, GB126_luc_, all tumors (N=21) were visualized on fluciclovine PET images. Across all subjects the median uptake ratio (SUVmax/SUVmean) for the normal contralateral brain was 1.2 (range 1.1 to 1.4).

**Figure 2:**
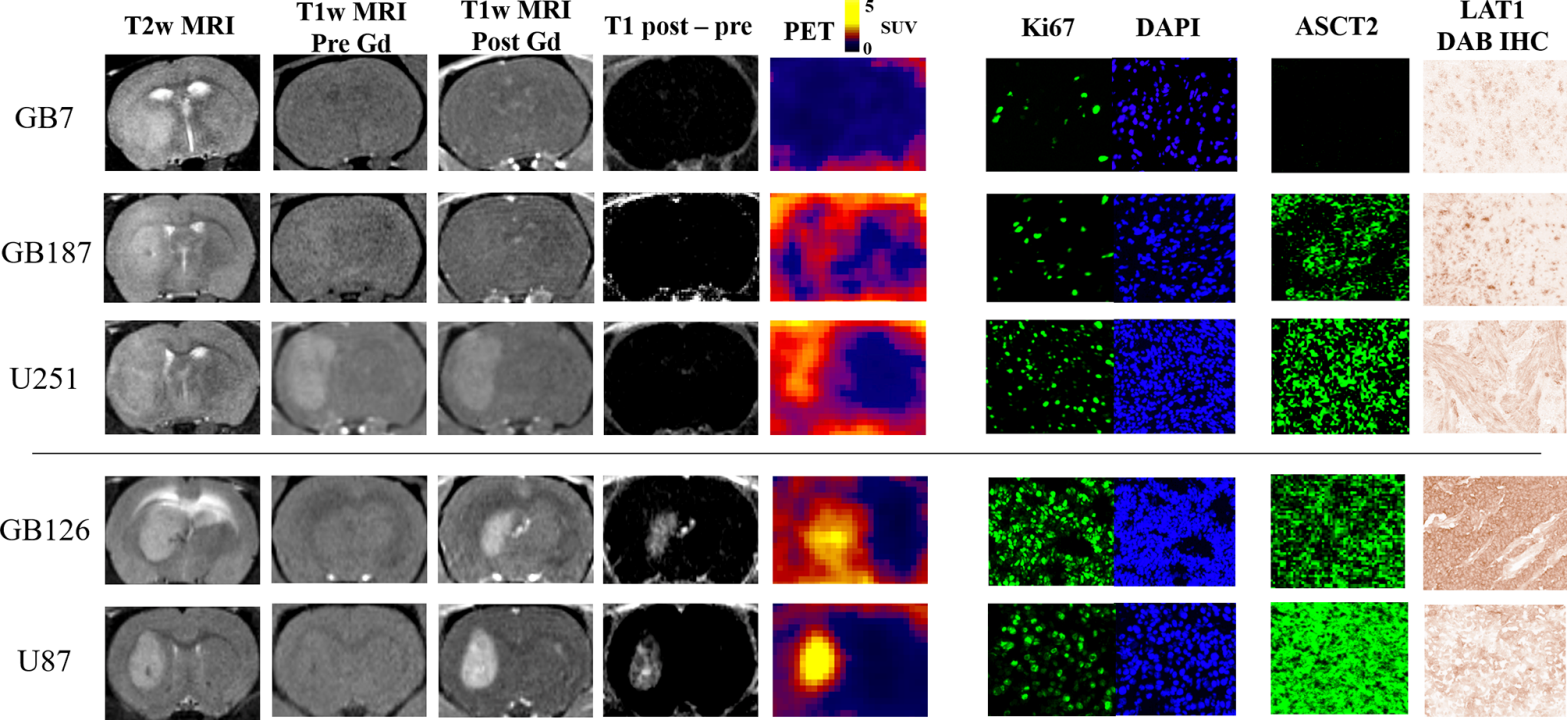
MRI and PET images shown alongside representative microscopy images of Ki67, ASCT2, and LAT1 staining. The tumors without gadolinium enhancement (i.e., top three rows; GB7_luc_, GB187_luc_, and U251_luc_) have lower PET uptake than the tumors with enhancement (i.e., bottom two rows; GB126_luc_ and U87_luc_). Within either the gadolinium enhancing or non-enhancing groups, tumors with higher ASCT2 demonstrated higher PET uptake.

**Figure 3:**
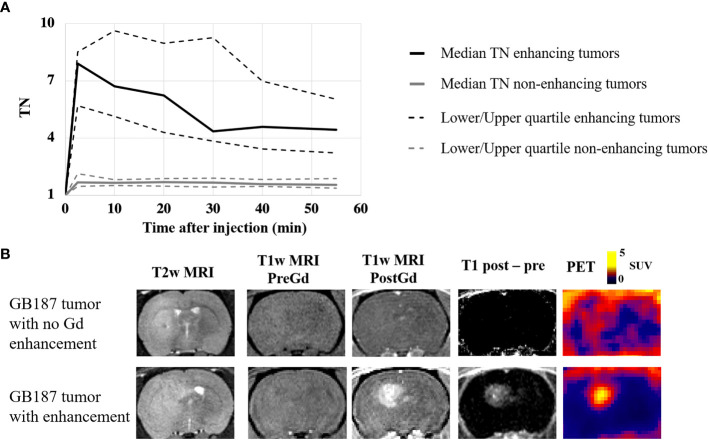
**(A)** PET uptake curves showing the median tumor-to-normal brain uptake ratio (TN) for gadolinium enhancing and non-enhancing tumors in the study. Significantly greater PET uptake was found in the enhancing tumors at all timepoints. **(B)** Example coronal image slices for a rat with a GB187_luc_ non-enhancing tumor (top row) and a rat with a GB187_luc_ enhancing tumor (bottom row). The PET images were averaged frames for the time 15-35 minutes after injection. PET uptake is evident in both tumors, but the enhancing tumor has greater uptake than the non-enhancing tumor.

**Figure 2** visually summarizes the imaging and histology findings for each tumor line. All tumors had LAT1 and Ki67 positive cells, and the fractions of positive cells were dependent on the tumor line. Cell density varied greatly across the tumor lines with GB126 and GB7 tumors having the highest and lowest cell densities, respectively. All U251_luc_ and GB7_luc_ tumors had no gadolinium enhancement on T1W MRI. All U87_luc_ and GB126_luc_ tumors had gadolinium enhancement on T1W MRI. For GB187_luc_ tumors, 25% (2/8) had gadolinium enhancement, while the remaining 75% (6/8) had no gadolinium enhancement. Those tumors with gadolinium enhancement had greater PET uptake than tumors without gadolinium enhancement (**Figure 3A**). For example, the TN_15-35_ for enhancing tumors was significantly greater than that for non-enhancing tumors with median values of 5.1 and 1.6 respectively (P<0.001). The increased uptake in tumor areas with gadolinium enhancement was also visually apparent on the PET images (**Figure 3B**). All gadolinium enhancing tumors could be visualized on fluciclovine PET images (N=17). For the non-enhancing tumors, 63% (10/16) could be visualized on fluciclovine PET images.

**Figure 4:**
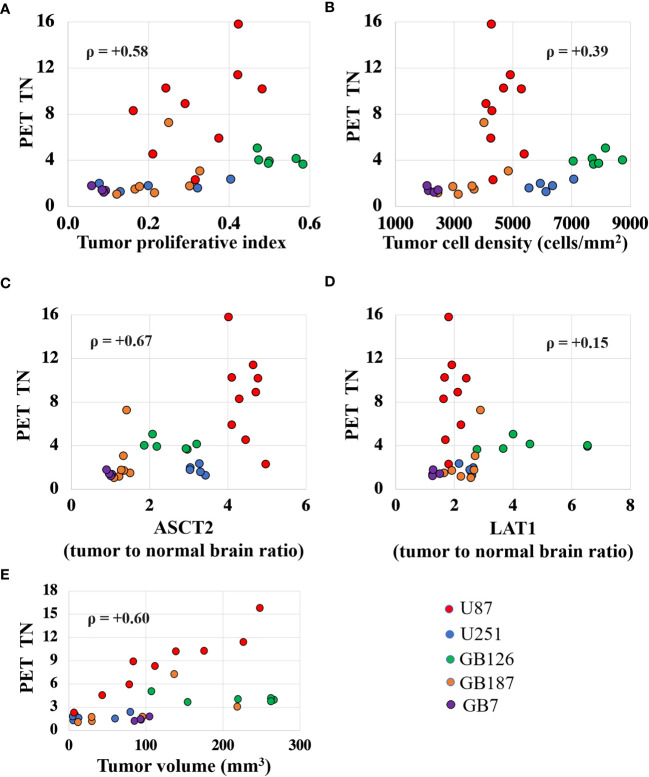
Scatter plots showing the univariate relationship between the PET tumor-to-normal brain uptake ratio (TN_15-35_) and the proliferative index **(A)**, cell density **(B)**, ASCT2 level **(C)**, LAT1 level **(D)**, and tumor volume **(E)**. Each data point represents measurements summarized for a single tumor and the color indicates the tumor line. The ρ value is the Spearman correlation coefficient.

**Figure 4** shows scatter plots that reveal the univariate relationship between fluciclovine TN_15-35_ and the various biologic measurements. Tumor fluciclovine uptake had a moderate positive correlation with tumor cell proliferation (**Figure 4A**), ASCT2 level (**Figure 4C**), and tumor volume (**Figure 4E**). Tumor fluciclovine uptake had a weak positive correlation with tumor cell density (**Figure 4** **B**) and LAT1 level (**Figure 4D**). The correlation amongst all histology measurements is shown in **Supplementary Table S1**.

**Figure 5:**
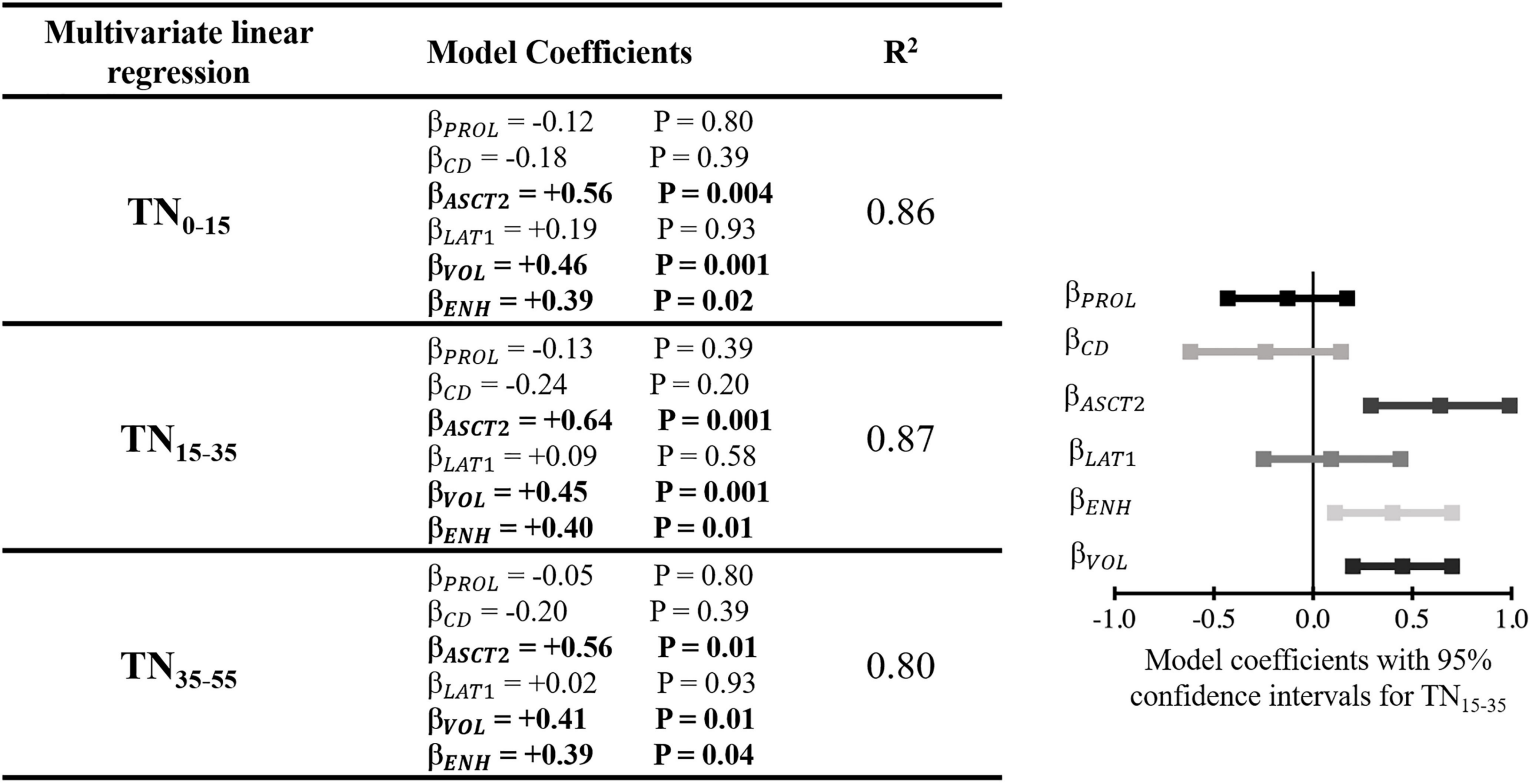
Left) Table showing the multivariate linear regression results with TN as the dependent variable. TN was extracted at three different timepoints after injection and each was included in a separate multivariate linear regression model (indicated by the different rows in the table). Right) Model coefficients, including confidence intervals, for the fit estimating TN_15-35_. This qualitatively demonstrates the effect size of variable included within the model. β model coefficient for a given variable; PROL, Ki67 proliferative index; CD, cell density; ENH, enhancing tumor; VOL, tumor volume.

**Figure 5** shows the multivariate linear regressions relating fluciclovine TN to the various pathology measurements. The regression model relating TN_15-35_ to the various pathology measurements demonstrated the highest R^2^ value of 0.87 relative to the TN values extracted at other timepoints. The lowest R^2^ value of 0.80 was found for TN_35-55_. Of the biologic measurements that were significantly associated with fluciclovine TN in the multivariate models, ASCT2 was the strongest (P=0.001), followed by tumor volume (P=0.001), and gadolinium enhancement status (P=0.01).

**Figure 6:**
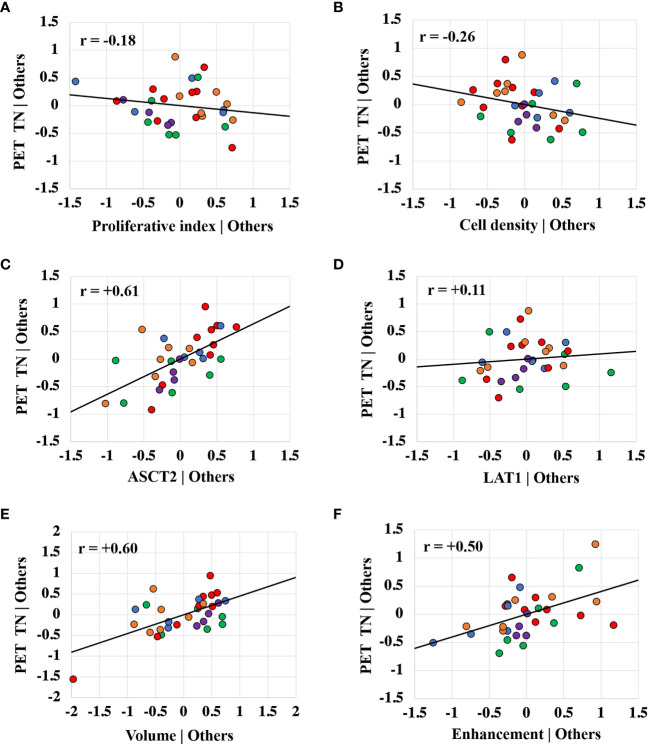
Partial regression plots showing the linear relationship between the PET tumor-to-normal brain uptake ratio (TN_15-35_) and the proliferative index **(A)**, cell density **(B)**, ASCT2 level **(C)**, LAT1 level **(D)**, tumor volume **(E)**, and gadolinium enhancement status **(F)**. Each data point represents measurements summarized for a single tumor and the color indicates the tumor line. The r value is the Pearson correlation coefficient. The linear regression line has intercept of zero and a slope equal to the multivariate linear regression coefficient tabulated in **Figure 4**. The vertical axis in these plots represents the calculated residuals from a linear regression model with the PET uptake as a function of all other independent variables except the independent variable of interest. The horizontal axis represents the calculated residuals from a linear regression model with the independent variable of interest as a function of all the other independent variables.

**Figure 6** shows partial regression scatter plots that indicate the partial correlation between TN_15-35_ and a given variable when taking into consideration the measurements of the other variables. These plots show that TN_15-35_ was most strongly correlated with tumor ASCT2 levels. Positive correlations were also found between TN_15-35_ and tumor volume, gadolinium enhancement status, and LAT1 levels. The partial regression plots show a weak negative correlation between fluciclovine TN_15-35_ and tumor cell proliferation and cell density. Similar trends were found for the TN values extracted at other timepoints.

## Discussion

Conventional MRI of glioblastoma provides qualitative assessments of tumor-induced edema and blood-brain barrier breakdown. Amino acid PET imaging enables regional assessment of tumor metabolic features that could complement conventional MRI but the biological basis of enhanced tracer uptake in the setting of brain tumors is still insufficiently characterized, which confounds clinical interpretation and application. This study sought to partially address this gap by correlating multiple biologic quantities with fluciclovine PET uptake across a range of human glioblastoma tumor models.

Fluciclovine PET uptake curves varied greatly across the five glioblastoma models assessed in this study. In general, the PET data could be split into two groups based on blood-brain barrier status (as assessed with gadolinium enhanced T1W MRI). The tumors with blood-brain barrier breakdown had rapid increases in fluciclovine uptake that peaked early within the first ten minutes after injection (primarily the U87_luc_ and G126_luc_ tumors). The tumors with intact blood-brain barrier (primarily U251_luc_, GB7_luc_, and GB187_luc_) had a relatively slow increase in fluciclovine uptake that continued throughout the fifty-five-minute uptake period. Interestingly, the commonly used amino acid PET uptake metric, the tumor-to-normal brain uptake ratio, peaked for all tumor models (median values) within the first thirty-five minutes. While tumors with intact blood-brain barrier (i.e., no gadolinium enhancement on T1W MRI) had relatively lower fluciclovine uptake, in most cases, the tumor uptake was still higher than that found in contralateral (normal) brain, indicating active blood-brain barrier transport and a potential role for leveraging fluciclovine PET to detect invading tumor regions in non-enhancing disease. However, of the non-enhancing tumors, 37% (6/16) were not visible on fluciclovine PET images, indicating that some non-enhancing tumors may not be detectable with fluciclovine PET. These six tumors that were not visible on fluciclovine PET images also had the six lowest ASCT2 intensities out of all 31 tumors assessed.

The fluciclovine PET tumor-to-normal brain uptake ratio values were significantly higher for tumors with blood-brain barrier breakdown than tumors without blood-brain barrier breakdown. These findings are similar to prior work showing fluciclovine TN was approximately twice as high in rat tumor models with blood-brain barrier breakdown relative to those without blood-brain barrier breakdown (26). Similar results have been found in human glioma patients, where tumors with gadolinium enhancement had at least twice as much fluciclovine uptake relative to tumors without gadolinium enhancement (16). These results support the notion that the degree of fluciclovine uptake correlates with blood-brain barrier status. The association between fluciclovine uptake and blood-brain barrier status remained significant even after accounting for other biologic variables in our multivariate model. This suggests fluciclovine uptake is not only correlated with blood-brain barrier status but also dependent on it. This could be explained by the fact that the initial extravasation of fluciclovine is increased in areas of blood-brain barrier breakdown, and this in turn leads to a greater pool of fluciclovine in the extracellular extravascular space that is available for uptake into tumor cells. It is interesting that the correlation between fluciclovine uptake and blood-brain barrier status remained significant in the multivariate model even at late timepoints after fluciclovine injection, suggesting the degree of fluciclovine uptake remains dependent on the tumor blood-brain barrier at relatively late timepoints after injection. These results indicate the blood-brain barrier status should be considered when interpreting fluciclovine PET measurements in the brain. For example, optimal analyses (e.g., thresholding, cut-off values, etc.) of fluciclovine PET images are likely going to be different for invasive non-enhancing glioblastoma regions than for enhancing glioblastoma regions. One limitation of this work is that although T1W gadolinium enhanced MRI is widely used clinically for assessing blood-brain barrier breakdown, there are other methods that are more sensitive to changes in the blood-brain barrier that could provide more accurate results (27, 28).

The multivariate model indicated the biologic variable with the strongest correlation to fluciclovine uptake was tumor ASCT2 levels. LAT1 levels were not significantly correlated with fluciclovine uptake in either the univariate or multivariate analysis. Prior cell culture studies indicated that in normal and alkaline pH conditions, ASCT2 is the primary transporter of fluciclovine into human brain tumor cells, whereas in acidic pH conditions, LAT1 and ASCT2 both contribute to fluciclovine transport (18). The results presented here support ASCT2 as the primary transporter of fluciclovine uptake into human glioblastoma *in vivo*. The lack of correlation of fluciclovine uptake with LAT1 could be because the tumor microenvironments were not sufficiently acidic to cause significant LAT1 transport of fluciclovine. Regardless, the correlation between fluciclovine uptake and ASCT2 supports the notion that within these glioblastoma models, fluciclovine PET can provide reliable quantification of tumor metabolism (or more specifically, uptake of amino acids mediated by high-affinity glutamine transporter ASCT2). Interestingly, the correlation of fluciclovine PET uptake with ASCT2 is different from other widely used amino acid radiotracers (FET and MET) whose PET uptake correlates primarily with LAT1 (13, 18, 29). This indicates fluciclovine PET measurements offer a distinct measure of glioblastoma amino acid metabolism that is not provided by other imaging modalities. These measurements could complement conventional anatomic MRI scanning and aide in brain tumor diagnosis, treatment planning, and therapy response assessment. However, tumors with low ASCT2 levels and an intact blood-brain barrier, may be difficult to detect with fluciclovine PET as was the case with the GB7_luc_ tumor line in this study, which did exhibit radiographic signs of tumor associated edema on T2 weighted MRI. In addition, due to differences in radiotracer affinity for LAT1 and ASCT2, the results from prior studies assessing other amino acid PET radiotracers may not necessarily apply to fluciclovine. Thus, fluciclovine studies cannot rely on preliminary data from other amino acid PET agents to establish utility.

Fluciclovine PET uptake showed a moderate positive correlation with tumor cell proliferation in the univariate analysis. This agrees with prior studies that showed a univariate correlation between fluciclovine uptake and brain tumor cell proliferation (30, 31). However, after accounting for other biologic variables in the multivariate analysis, we found no significant correlation between fluciclovine uptake and cell proliferation. A similar result was found for the relationship between fluciclovine uptake and tumor cell density. Tumor volume was significantly correlated with fluciclovine PET uptake in both the univariate and multivariate analysis. This is likely due to partial volume effects that are inherent to the PET scanner acquisition and these results indicate the importance of considering these effects in the analysis of fluciclovine PET brain tumor images (32).

## Data Availability Statement

The raw data supporting the conclusions of this article will be made available by the authors, without undue reservation.

## Ethics Statement

The animal study was reviewed and approved by St. Joseph Hospital and Medical Center’s Institutional Animal Care and Use Committee.

## Author Contributions

CQ acquired funding for the study. CQ and SM designed the studies and provided guidance during the studies. MS and DH conducted the studies. MS wrote the manuscript.All authors contributed to the article and approved the submitted version.

## Funding

We acknowledge the generosity of our sponsors for funding this work, including the Barrow Neurological Foundation, the Arizona Biomedical Research Centre (ADHS18-198850), Students Supporting Brain Tumor Research, and Blue Earth Diagnostics Ltd. This study received the Fluciclovine tracer from Blue Earth Diagnostics Ltd.

## Conflict of Interest

The authors declare that the research was conducted in the absence of any commercial or financial relationships that could be construed as a potential conflict of interest.

The authors declare that this study received funding from Blue Earth Diagnostics Ltd. The funder had the following involvement with the study: edits to the final version of the manuscript.

## Publisher’s Note

All claims expressed in this article are solely those of the authors and do not necessarily represent those of their affiliated organizations, or those of the publisher, the editors and the reviewers. Any product that may be evaluated in this article, or claim that may be made by its manufacturer, is not guaranteed or endorsed by the publisher.
