# Supplementary material for: Imaging Glioblastoma With 18F-Fluciclovine Amino Acid Positron Emission Tomography
Source: Front Oncol. 2022 Jan 31;12:829050. doi: 10.3389/fonc.2022.829050 (PMC8841434; doi:10.3389/fonc.2022.829050)
Supplement: Supplementary file 1 [file Table_1.docx]

**Table S1:** *Spearman correlation coefficients for various measurements (N=31).*


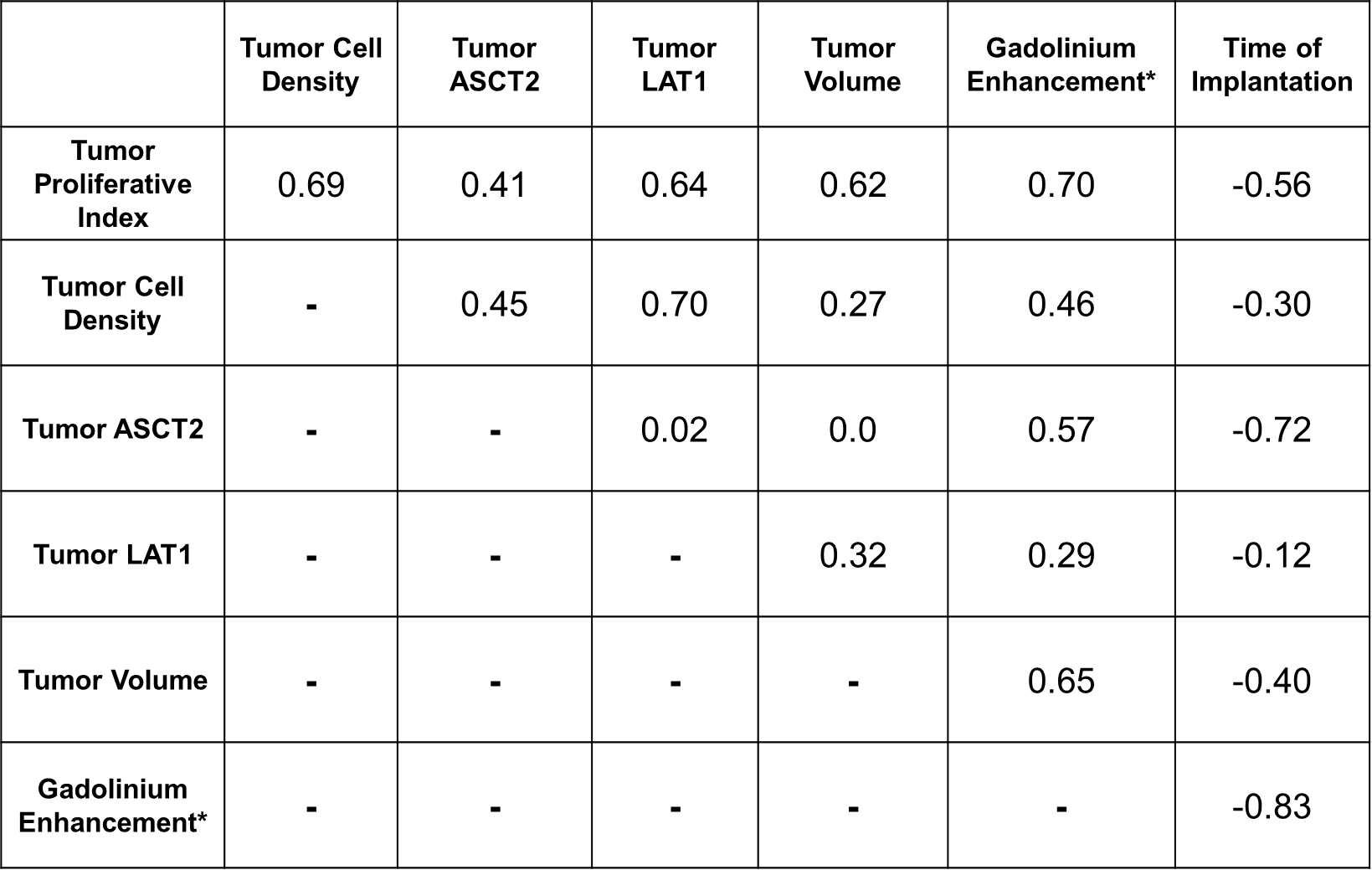


*Gadolinium enhancement was included in calculations with numerical value of 1 (representing no tumor enhancement) or 2 (representing tumor enhancement).
